# Neonatal microbiota development and the effect of early life antibiotics are determined by two distinct settler types

**DOI:** 10.1371/journal.pone.0228133

**Published:** 2020-02-05

**Authors:** Anat Eck, Nicole B. M. M. Rutten, Maartje M. J. Singendonk, Ger T. Rijkers, Paul H. M. Savelkoul, Clemens B. Meijssen, Clarissa E. Crijns, Johanna H. Oudshoorn, Andries E. Budding, Arine M. Vlieger

**Affiliations:** 1 Department of Medical Microbiology and Infection Control, Amsterdam UMC, VU University Amsterdam, Amsterdam, The Netherlands; 2 Department of Pediatrics, St Antonius Hospital, Nieuwegein, The Netherlands; 3 Department of Pediatric Gastroenterology and Nutrition, Emma Children’s Hospital, Amsterdam UMC, University of Amsterdam, Amsterdam, The Netherlands; 4 Department of Sciences, University College Roosevelt Academy, Middelburg, The Netherlands; 5 Department of Medical Microbiology, NUTRIM School of Nutrition and Translational Research in Metabolism, Maastricht University Medical Centre, Maastricht, The Netherlands; 6 Department of Pediatrics, Meander Medical Centre, Amersfoort, The Netherlands; 7 Department of Pediatrics, Tergooi Hospital, Blaricum, The Netherlands; 8 Department of Pediatrics, Gelre Hospitals, Apeldoorn, The Netherlands; University of Illinois, UNITED STATES

## Abstract

The neonatal period, during which the initial gut microbiota is acquired, is a critical phase. The healthy development of the infant’s microbiome can be interrupted by external perturbations, like antibiotics, which are associated with profound effects on the gut microbiome and various disorders later in life. The aim of this study was to investigate the development of intestinal microbiota and the effect of antibiotic exposure during the first three months of life in term infants. Fecal samples were collected from healthy infants and infants who received antibiotics in the first week of life at one week, one month, and three months after birth. Microbial composition was analyzed using IS-pro and compared between antibiotics-treated and untreated infants. In total, 98 infants, divided into four groups based on feeding type and delivery mode, were analyzed. At one week of age, samples clustered into two distinct groups, which were termed “settler types”, based on their Bacteroidetes abundance. Caesarean-born infants belonged to the low-Bacteroidetes settler type, but vaginally-born infants were divided between the two groups. The antibiotics effect was assessed within a subgroup of 45 infants, vaginally-born and exclusively breastfed, to minimize the effect of other confounders. Antibiotics administration resulted in lower Bacteroidetes diversity and/or a delay in Bacteroidetes colonization, which persisted for three months, and in a differential development of the microbiota. Antibiotics resulted in pronounced effects on the Bacteroidetes composition and dynamics. Finally, we hypothesize that stratification of children’s cohorts based on settler types may reveal group effects that might otherwise be masked.

## Introduction

Infants develop an initial microbiome from birth onwards, ultimately becoming colonized with a wide variety of microorganisms.[1–3] The gut microbiota development continues during the first years of life, a critical period for development and maturation of the immune system, in which the foundation for future health is laid.[4] Various factors influence the intestinal microbiota and its establishment, including mode of delivery, dietary patterns, genetic factors and possibly administration of pro-, pre- and antibiotics.[5–12]

In vaginally delivered infants, colonization with maternal vaginal and fecal microbes, including lactobacilli, group B streptococci, and certain bifidobacterial species, strongly suggests a maternal signature.[12–14] Gut microbiota of caesarean-born infants resembles maternal skin microbes (staphylococci),[11, 15, 16] and harbors less *Bifidobacterium*, *Escherichia coli* and *Bacteroides* species compared to vaginally-born children,[4, 13] which may explain the higher incidence of immune-mediated diseases in these children.[1, 17]

Healthy microbiome development can be interrupted by external perturbations, like antibiotics. While the use of perinatal broad-spectrum antibiotics has become common in modern obstetric and neonatal practice, evidence is increasing that exposure to antibiotics in early life is associated with profound effects on the gut microbiome and various disorders later in life, like atopy, inflammatory bowel disease (IBD), diabetes and obesity.[13–21] Recent epidemiological and mechanistic data on the association between early antibiotic use, dysbiosis and disease, support this concern.[19, 20, 22]

While culture-dependent techniques already revealed an altered intestinal microbiota in antibiotic-treated infants,[23, 24] molecular approaches dramatically refined the perspective on gut microbiota composition and dynamics, [25, 26] and consistently indicate that early life antibiotic exposure has pervasive effects on microbial colonization and development. Lower bacterial diversity was observed in preterm infants, who routinely receive empiric antibiotic therapy, compared to untreated (term) infants.[27] In addition, a longitudinal study, including monthly sampling during the first 36 months, revealed that the microbiota of antibiotic-treated children was less stable and less diverse at both species and strains levels.[28] Moreover, while beneficial species, like *Eubacterium rectale* and bifidobacteria species, were found in lower abundance in antibiotic-treated children compared to untreated children,[7, 9, 28, 29] unusual colonization of potentially multidrug-resistant members, such as members of Enterobacteriaceae and *Enterococcus*, was observed in the gut microbiota of infants following antibiotic treatment.[27, 29, 30] Finally, a recent prospective study described long-term differences in gut microbiome composition of newborn infants who were exposed to antibiotics, as well as emergence of antibacterial resistance genes.[31]

This study aims to investigate the development of intestinal microbiota during the first three months of life in term infants and the effect of neonatal antibiotic exposure on this development. Antibiotics-induced effects in infants are not yet fully understood, leaving an important gap in understanding of how they shape the microbiome during this important developmental window and how the ecological balance can be restored.

## Materials and methods

### Study design

Subjects were recruited from the maternity wards of four teaching hospitals in the Netherlands: the St. Antonius Hospital in Nieuwegein, the Tergooi Hospital in Blaricum, the Gelre Hospitals in Apeldoorn and the Meander Medical Centre in Amersfoort. Patients were enrolled between January 2012 and February 2014. Parents of term-born infants (≥ 36 weeks of gestational age) who stayed in the hospital >24 hours were approached for participation in the study. Exclusion criteria were: 1. Congenital illness or malformations; 2. Severe perinatal infections for which transfer to the neonatal intensive care unit was needed; 3. Maternal probiotic use within six weeks before delivery; and 4. Insufficient knowledge of the Dutch language. Written informed consent (on behalf of the children) was obtained from parents at enrolment. This study was approved by the joint Medical Ethics Committee (VCMO, nowadays MEC-U: Medical Research Ethics Committees United (Nieuwegein)) and a proof of local feasibility was given for all participating centers. The study was performed in accordance with the ethical standards laid down in the 1964 Declaration of Helsinki and its later amendments and was part of a larger prospective cohort study on the influence of antibiotics on microbiota and disease (INCA study, registered on ClinicalTrials.gov: NCT02536560).

Infants were divided into four groups based on feeding type during the first three months and delivery mode: A] vaginally-born & formula-fed (VF), B] caesarean section & formula-fed (CF), C] caesarean section & breastfed (CB), and D] vaginally-born & breastfed (VB). The last group was divided into two subgroups according to antibiotic treatment. One subgroup included infants who received seven days of antibiotic treatment in the first week of life according to the local hospital protocols (VB+AB), while the other subgroup included infants who did not receive any antibiotics (VB-AB). The latter was considered as a control group, to minimize the effect of confounders like feeding type and delivery mode.

### Stool collection

Maternal stool samples (M) were caught in a tray within one week after delivery. Infants’ stool samples were collected from diapers at day seven (T = 1), and at the end of the first month (T = 2) and third month (T = 3). These were transferred to stool collection vials and immediately frozen in the hospital or home freezers (-20°C). Frozen samples collected at home were transported on ice to the hospital and immediately stored at -20°C.

### Bacterial DNA isolation

A fecal sample (100–400 mg) was placed in an Eppendorf container. Then, a 200 μl (for 100 mg fecal input)– 800 μl (for 400 mg fecal input) suspension was made in nucliSENS lysis buffer (provided for easyMAG DNA isolation, an automated system for total nucleic acid extraction, bioMérieux Clinical Diagnostics, Marcy l'Etoile, France), which was subsequently vortexed for 1 minute, shaken for 5 minutes, and centrifuged at 16.2 *g* for 2 minutes at room temperature. Supernatant (100 μl) was transferred to an 8-welled easyMAG container, and another 2 ml nucliSENS lysis buffer was added. After incubation at room temperature for ≥10 min, 70 μl of magnetic silica beads was added. The mixture was placed in the easyMAG machine and the “specific A” protocol was chosen, selecting the off-board workflow and finally eluting DNA in 110 μl of elution buffer. Isolated fecal DNA was stored at 4°C and diluted 10-fold before use in PCR, which was performed within hours.

### 16S-23S IS profiling of gut microbiota

Amplification of interspace (IS) fragments was performed with IS-pro (IS-diagnostics, Amsterdam, The Netherlands), which combines bacterial species differentiation by the length of 16S-23S rRNA IS regions with instant taxonomic classification by phylum-specific fluorescently-labeled PCR primers. The procedure includes two multiplex PCRs, providing a broad coverage for Firmicutes, Actinobacteria, Fusobacteria and Verrucomicrobia (FAFV), Bacteroidetes and Proteobacteria.[32] Amplifications were performed on a GeneAmp PCR system 9700 (Applied Biosystems, Foster City, CA). PCR conditions were: 10 cycles of 94°C for 30s, 67°C to 57°C (‘touch down’) for 45s, 72°C for 1 min; 25 cycles of 94°C for 30s, 57°C for 45s, and 72°C for 1 min; 72°C for 11 min and a final extension at 4°C. Each PCR mixture (25 μl) contained 10μl of buffered DNA, 1x superTaq buffer (Applied Biosystems), 200μM deoxynucleoside triphosphates, 0.04% BSA, 1 U of superTaq (Applied Biosystems), and 0.13 μM of each primer.

### Data analysis

After pre-processing (IS-pro software suite, IS-diagnostics, Amsterdam, The Netherlands), each sample was represented as a microbial profile, consisting of color-labeled peaks. Each peak represented a specific IS fragment (measured as nucleotide length) and a color related to a specific phylum group. Each peak was designated as an operational taxonomic unit (OTU) and its corresponding intensity, reflecting the relative quantity of PCR product (measured in relative fluorescence units (RFU)), as abundance. Intensity values were log2 transformed in order to compact the range of variation in peak heights and to reduce the impact of dominant peaks. A clustered heat map was generated based on a cosine correlations matrix of all profile data, followed by clustering with the unweight pair group method with arithmetic mean (UPGMA).

Alpha diversity was calculated using the Shannon index using the vegan package in R (Foundation for Statistical Computing, Vienna, Austria) and SPSS (Chicago, IL, USA).[33] Differences were tested with Mann-Whitney *U* test. Dissimilarities between samples were calculated using cosine distance between pairs of profiles. A lower cosine value indicates that two profiles are more similar to each other. Data are shown as median (interquartile range) for continuous variables. Differences between groups and potentially confounding variables were tested for independence by chi-square test and were considered to be significant for p<0.05.

## Results

### Study population characteristics

In total, 98 infants, divided into four groups based on feeding type and delivery mode, were analyzed. The effect of antibiotics was assessed by comparing the VB+AB group to VB-AB group (n = 45). Participant characteristics are shown in Table 1. Baseline characteristics were not statistically different between the groups (compared by ANOVA).

**Table 1:** Study cohort characteristics.

INFANTS		VB+AB (n = 21)	VB-AB (n = 24)	VF (n = 21)	CF (n = 24)	CB (n = 8)
	Male	10 (48%)	10 (42%)	12 (57%)	12 (50%)	2 (25%)
	Mean gestational age	40 wks, 2 days	39 wks, 1 day	39 wks, 5 days	39 wks, 6 days	39 wks, 6 days
	Birth weight in gram (SD)	3768 (556)	3423 (463)	3381 (430)	3995 (647)	3597 (500)
MOTHERS	Age in years (SD)	No maternal samples analysed	32.6^a^ (3,7)	32.9 (4.8)	32.3 (4.1)	No maternal samples analysed
	Peripartum antibiotics (yes/no/unknown)	4 / 16 / 1	3 / 8 / 13	Missing data	Missing data	Missing data
	C-section indication (planned/emergency)				15 / 9	4 / 4

AB: antibiotic treatment group; VB: no antibiotics, vaginally-born, breastfed; VF: no antibiotics, vaginally born, formula fed; CF: no antibiotics, caesarean-born, formula fed; CB: no antibiotics, caesarean-born, breastfed.

^a^ data available for n = 23 mothers.

One infant in the VB-AB received antibiotic treatment shortly before the third sample, which was therefore excluded. Participating infants in the VB+AB group received intravenous antibiotics for a period of seven days, because of a high suspicion of neonatal sepsis due to overt clinical signs of infection and/or an increased serum level of C-reactive protein. Blood cultures were negative in all VB+AB children after 48 hours. The majority of VB+AB infants received a standardized combination scheme of seven days penicillin combined with gentamycin for the first two days (n = 11, 52%) or amoxicillin (n = 8, 38%) instead of penicillin. The other two prescribed schemes were amoxicillin with ceftazidime (a third-generation cephalosporin) and amoxicillin/clavulanic acid with gentamycin. Since only a few infants received an antibiotic scheme different from the predominant schemes, we were unable to test for effects of different regimens and considered all VB+AB infants as one group. While maternal antibiotic treatment may also affect the newborn, [13] our related information was incomplete and did not allow a comprehensive analysis in regard to this factor.

### Two distinct clusters

**Fig 1:**
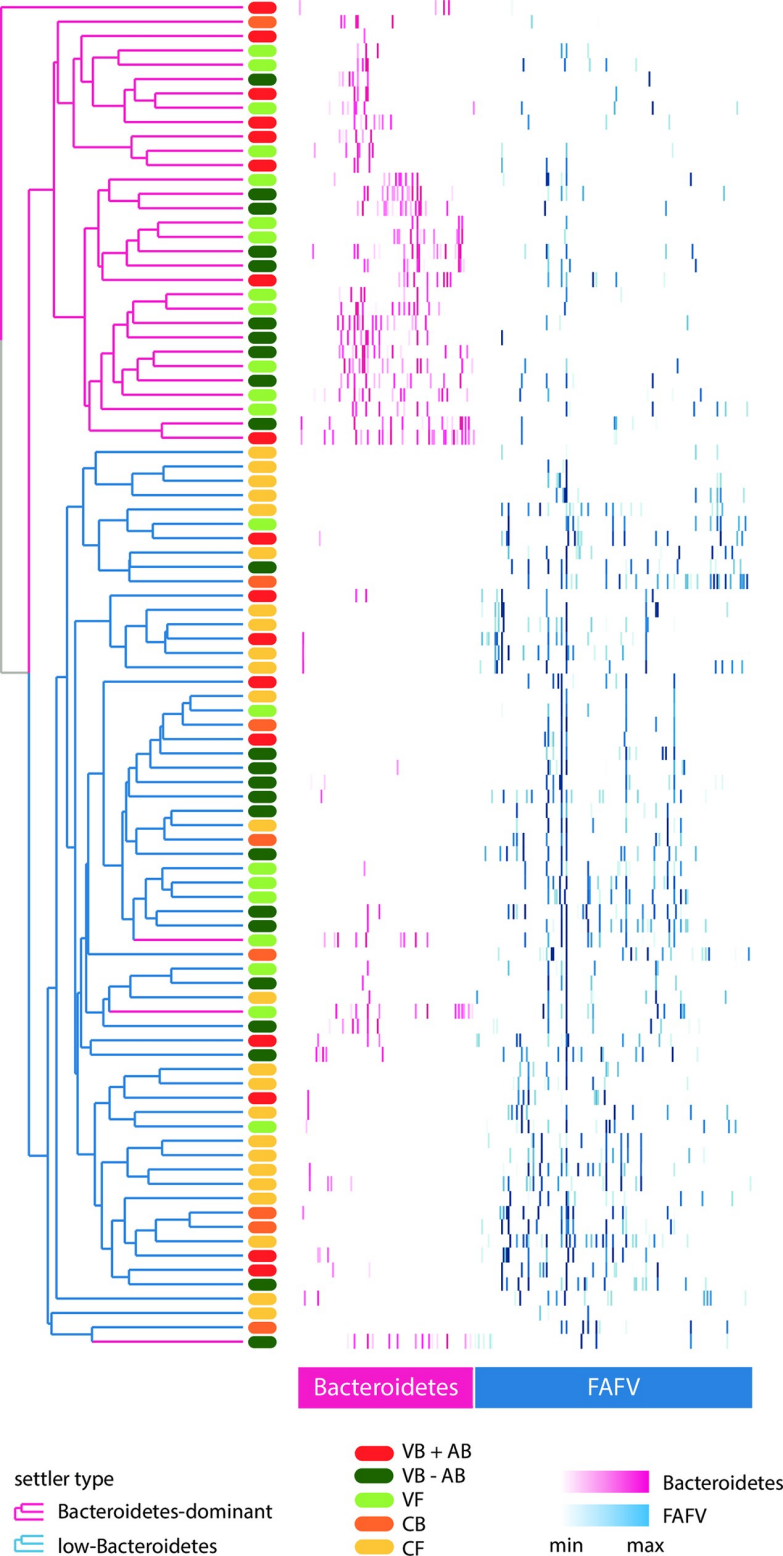
Heat map of all profiles at T = 1 (one week), sorted and colored by phylum. Abundance of OTUs in each sample at T = 1 (one week). Rows correspond to samples (antibiotic treatment: red; vaginal delivery: green; caesarean section: orange); columns correspond to OTUs. Phyla color shades represent the abundance of each OTU in a sample (Bacteroidetes: pink; FAFV: blue). Cosine correlations and hierarchical clustering were calculated on the raw data, but for a better interpretation we only present the called peaks in this visualization. Top cluster samples (pink) belong to the Bacteroidetes-dominant settler type. Bottom cluster samples (blue) belong to the low-Bacteroidetes settler type (with three exceptions). FAFV = Firmicutes, Actinobacteria, Fusobacteria, Verrucomicrobia; OTU = operational taxonomic units.

At one week of age, two distinct sub-populations were identified. Fig 1 shows the clustering of infants at T = 1, with one group characterized by Bacteroidetes-dominant microbiota and the other with a low Bacteroidetes microbiota. These clusters were not associated with infants’ antibiotic treatment, feeding mode, maternal GBS carriage or segregated by hospital (p = 1.0 for all), and therefore suggested distinct types of early microbial colonization, which we termed “settler types”.

Settler types were discriminated empirically at a cut-off of 30% relative abundance of Bacteroidetes (total Bacteroidetes signal divided by total bacterial signal). All Bacteroidetes-dominant samples came from children delivered vaginally, whereas all children delivered by caesarean section (except one) belonged to the low-Bacteroidetes settler type. However, a significant proportion (42%) of children delivered vaginally had a low-Bacteroidetes microbiota, similar to caesarean-born children (Table 2).

**Table 2:** Distribution of infants per settler type.

Settler type	VB+AB (n = 21)	VB-AB (n = 24)	VF (n = 21)	CF (n = 24)	CB (n = 8)
Bacteroidetes-dominant	9^a^	11	14	0	1
Low-Bacteroidetes	8	13^b^	7	24	7

^a^ Four infants were missing T = 1 sample.

^b^ Mothers of 2 infants received antibiotics peripartum.

AB: antibiotic treatment; VB: vaginally-born, breastfed; VF: vaginally born, formula fed; CF: caesarean-born, formula fed; CB: caesarean-born, breastfed.

The type of feeding, breast- or formula-feeding, did not result in significant differences at the phylum level (comparing VB-AB versus VF, and CB versus CF). This may have been due to the small group sizes and/or subtle differences that were not detected by the technique applied. Further analysis revealed that settler types were not determined by feeding type; the numbers of infants that were breast- or formula fed were distributed evenly between the Bacteroidetes-dominant and the low-Bacteroidetes settler types. However, in the latter, an effect of feeding type was shown, with breast-fed children harboring more *Staphylococcus epidermidis* compared to formula fed children. Formula-fed children acquired higher numbers of *Enterococcus faecalis* over time.

For a deeper view of the neonatal microbial composition, we determined the core species–found in >50% of children within each settler type. Infants of the Bacteroidetes-dominant group were largely colonized by *Bacteroides vulgatus* and *Parabacteroides distasonis*, common and abundant species in the human gastrointestinal tract.[34, 35] In the low-Bacteroidetes group, *Staphylococcus epidermidis* and *Streptococcus salivarius*, common members of oropharyngeal and skin microbiota, were the predominant species.

**Fig 2:**
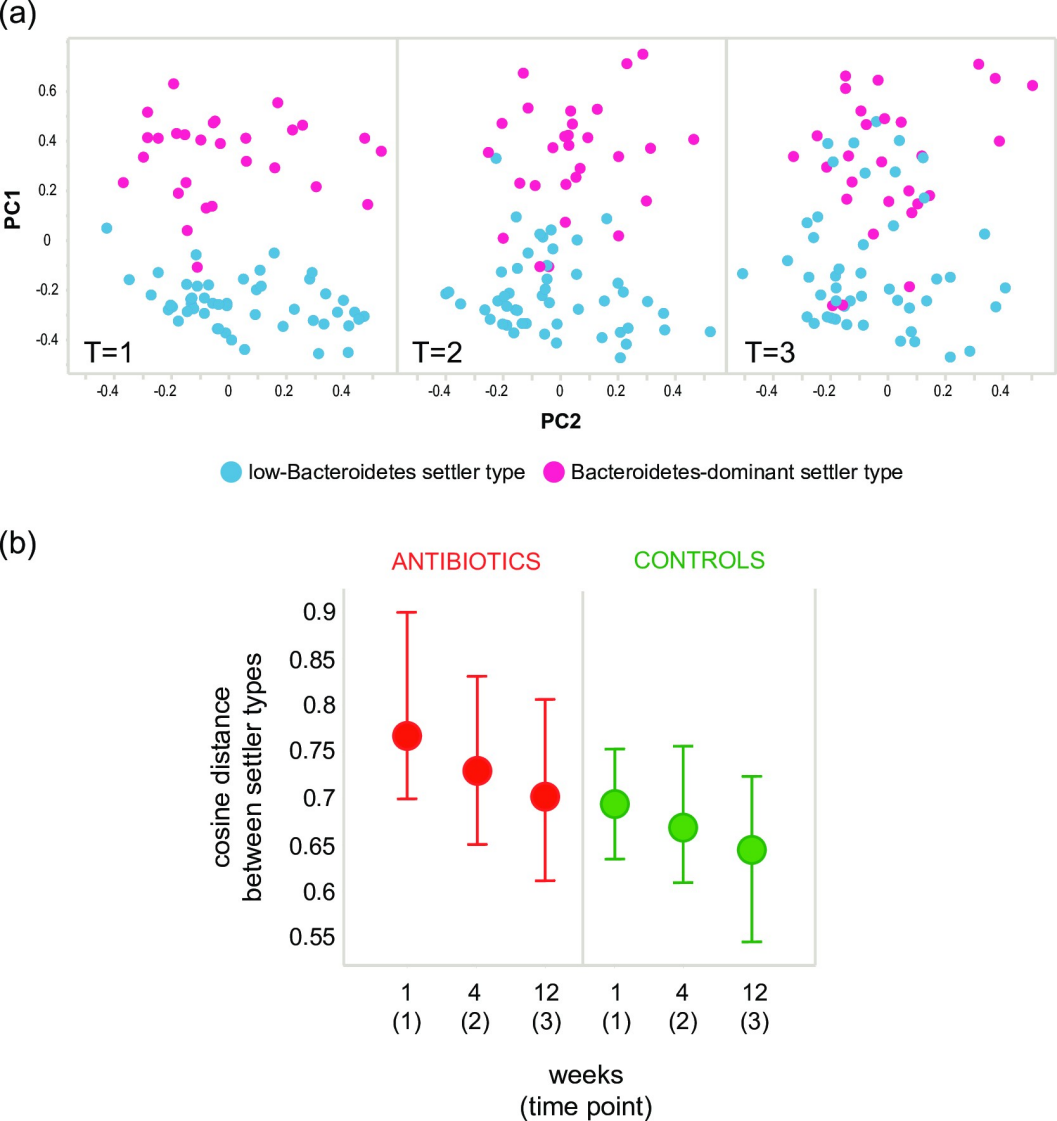
Convergence of settler types over time. A principle coordinate analysis of intestinal microbiota of all untreated infants at week 1 (T = 1), month 1 (T = 2) and month 3 (T = 3). Samples are colored by settler type. (b) Cosine distances, as a measure of community dissimilarity between settler types, displayed over time by treatment group. Dots indicate median cosine distances, bars indicate IQR (inter quartile range). A higher distance indicates the settler types are less similar.

To investigate the effect of settler types on early microbiota development, we portrayed the microbial composition at each time point per settler type in a principal coordinate analysis (PCoA) (Fig 2A). Segregation of samples according to settler type was most outspoken at T = 1, and, despite a convergence of microbiota profiles of different settler types, remained evident also at later time points. A delayed convergence of the clusters, reflected by higher cosine distances, was observed in children treated with antibiotics (Fig 2B; p<0.0001 at all time points).

Abundance and diversity of Bacteroidetes and FAFV phyla members differed significantly between settler types (p<0.005 at all time points), while Proteobacteria abundance and diversity were similar. In the low-Bacteroidetes settler type, Bacteroidetes abundance and diversity increased over time, whereas FAFV abundance and diversity decreased. At the same time, FAFV diversity increased in the Bacteroidetes-dominant settler type towards a value similar to that observed in the other group. Total diversity was significantly lower in the low-Bacteroidetes settler type at T = 2 and T = 3 (p<0.05).

Maternal stool samples (only available for the VB-AB, VF and CF groups) clustered separately from infants’ samples, with no segregation according to settler types. Candidates of maternal transmission of certain bacterial species, such as *B*. *vulgatus* and *Bacteroides fragilis*, were identified in individual cases of the Bacteroidetes-dominant settler type, but no common species between mother and infant were observed in the low-Bacteroidetes settler type (S1 Fig).

### Antibiotics effect on Bacteroidetes

**Fig 3:**
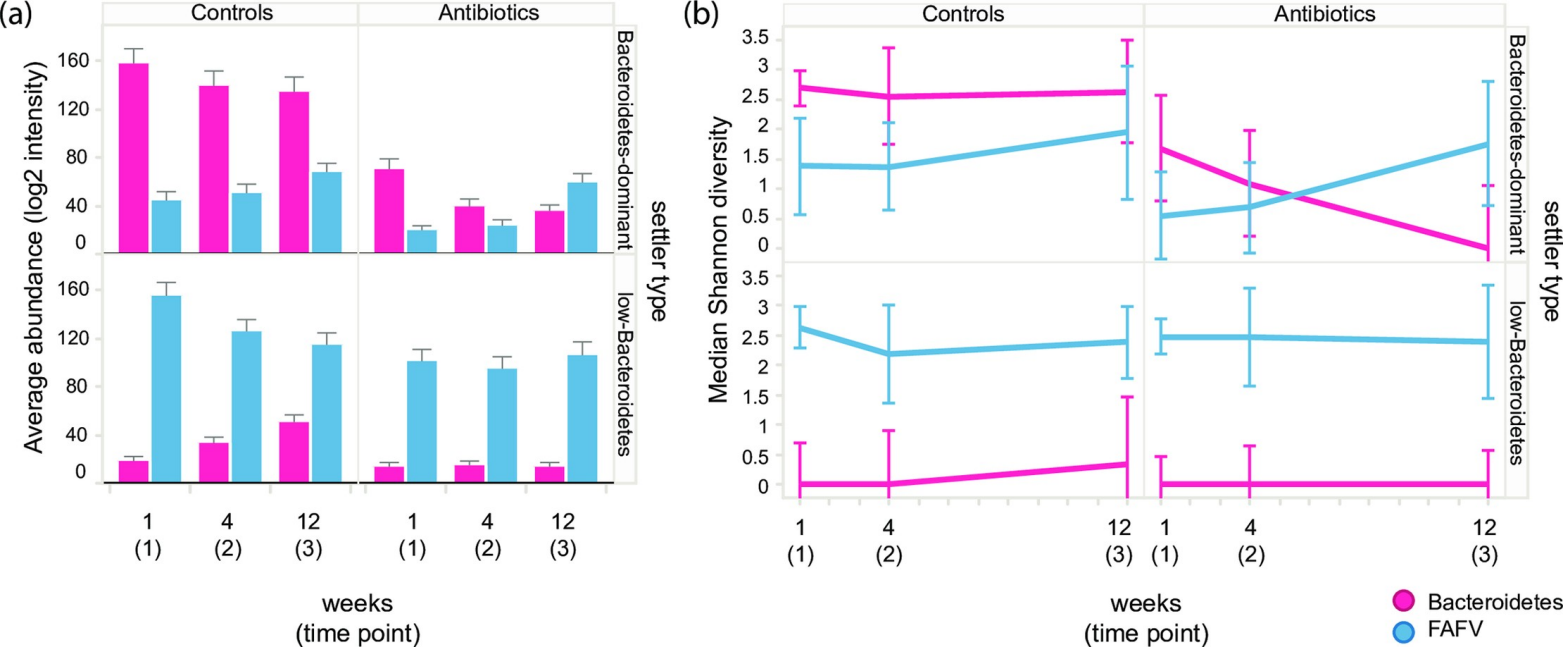
Abundance and diversity of Bacteroidetes and FAFV by settler type and treatment group. (a) Average abundance of Bacteroidetes and FAFV over time by settler type (rows) and treatment group (columns). Values are log2 transformed intensities (measured as log2 RFU). Error bars depict the standard error. (b) Median Shannon diversity index of Bacteroidetes and FAFV over time by settler type (rows) and treatment group (columns). Error bars denote the upper and lower IQR (inter quartile range); RFU—relative fluorescent units; FAFV = Firmicutes, Actinobacteria, Fusobacteria, Verrucomicrobia.

In the Bacteroidetes-dominant settler type, Bacteroidetes abundance and diversity were significantly lower at all time points in antibiotic-treated infants compared to controls (p = 0.03, p = 0.003 and p<0.001 for abundance, and p = 0.009, 0.004, 0.004 for diversity, at T = 1, T = 2 and T = 3 respectively; Fig 3). The impact on Bacteroidetes was still evident three months after cessation of antibiotics. In antibiotic-treated infants that belonged to the low-Bacteroidetes settler type, Bacteroidetes growth was inhibited throughout the whole study period.

### Antibiotics effect on microbial development

**Fig 4:**
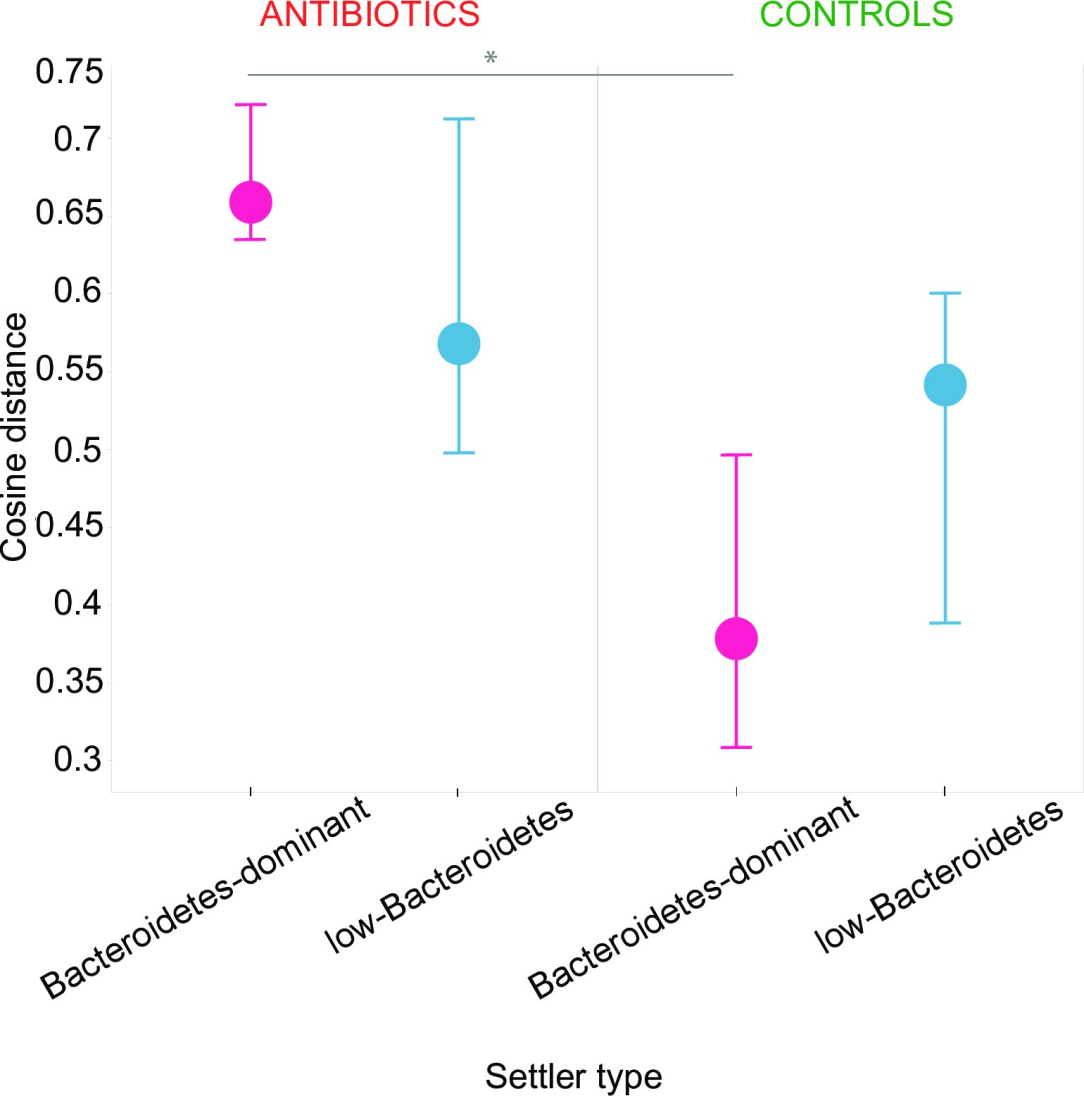
Cosine distances as a measure of community dissimilarity within individuals over time by settler type and treatment group. Cosine distances displayed by settler type in antibiotic treated infants (left) and controls (right). Distances shown are between one week of age (T = 1) and three months of age (T = 3) within each individual. Dots indicate median cosine distances (during the 3-month interval) of all individuals of each settler type; bars indicate IQR (inter quartile range).

Antibiotics longitudinal effects on the microbiota were assessed using cosine distances calculated between profiles of each infant for all possible combinations of time points. The microbial development of controls in the Bacteroidetes-dominant settler type was the most consistent, as can be seen by the lowest overall cosine distances, whereas antibiotic-treated infants of the same settler type had the most perturbed microbial development, reflected by the highest cosine distances (Fig 4; p<0.0001 at the displayed interval, T = 1–3).

## Discussion

We investigated the early development of gut microbiota and how it is affected by antibiotics. At one week of age, two distinct subgroups, characterized by different Bacteroidetes colonization patterns, were identified, regardless of treatment group. These groups were denoted accordingly Bacteroidetes-dominant settler type and low-Bacteroidetes settler type. Importantly, we found that almost all children born by caesarean section belonged to the latter, while only 58% of vaginally-born children belonged to the Bacteroidetes-dominant settler type.

Although the separation into clusters remained during the first three months, convergence was observed, mostly by acquisition of Bacteroidetes by infants in the low-Bacteroidetes settler type. However, while Bacteroidetes abundance and diversity increased over time, it did not reach the levels found in infants of the other settler type during this period.

A similar finding, depicting the presence of two clearly demarcated subgroups in vaginally-born infants, was previously described in two independent cohorts.[28, 36] It was first observed in a small group of seven term, vaginally delivered and exclusively breast-fed neonates, who differed by the levels of *Bacteroides* throughout the neonatal period.[38] More recently, it was again described in a longitudinal study, in which infants showed one of two microbial signatures based on the abundance of *Bacteroides* in the first six months of life.[28]

It has already been shown that vaginally delivered infants acquire bacterial communities resembling their own mother's vaginal microbiota.[37] However, the data presented in this study indicate that a substantial number (42%) of vaginally delivered children have an initial microbiota very similar to that of caesarean-born children, with an underrepresentation of Bacteroidetes members.[38] We hypothesize that delivery mode may not be the only influencing factor in settler type determination, and that variation in early Bacteroidetes acquisition among vaginally-delivered infants may be introduced as a result of exposure to maternal fecal microbiota and/or by the vaginal microbiota composition. Therefore, different conditions during labor and delivery may be important for initial microbiota acquisition, like duration of ruptured membranes, duration of delivery, maternal bowel movements during labor, and environment (home, hospital delivery room, operating room). These factors are usually discarded in most analyses but can potentially determine the settler type, which might be associated with certain conditions with which Caesarean section is inconclusively associated.

Antibiotic treatment resulted in a lower Bacteroidetes diversity and/or delayed Bacteroidetes colonization, which persisted for at least three months. Bacteroidetes members play an important role in the development of a healthy, stable gut microbiota. Lower Bacteroidetes abundance, found in Caesarean-born infants, was associated with an increased risk of developing type 1 diabetes, asthma and allergic diseases.[38–41] Furthermore, the predominant species of the Bacteroidetes-dominant settler type we identified were previously found as prevalent members of a healthy core microbiota in children, while missing from that of pediatric IBD patients.[42] While the mechanisms of these associations are yet to be elucidated, it is important to bring into consideration that a perturbed microbial composition in early life may have long term consequences. For example, children that were characterized by a low-*Bacteroides* signature at the first six months also presented lower overall microbial diversity that persisted even at 36 months, when *Bacteroides* had become highly abundant in this group.[28]

In both treatment groups, a positive correlation was observed between the time span and cosine distances calculated between profiles. This is naturally due to the continuous increase in richness during infancy. However, microbial profiles of antibiotic-treated infants of both settler types (although not significant for the low-Bacteroidetes settler type) diverged more over three months, suggesting a differential microbiota development and a more aberrant colonization pattern in this group. For example, *E*. *coli* was less prevalent in antibiotic-treated infants and we observed a shorter and a more fluctuating colonization compared to control infants; only four infants continuously carried *E*. *coli* at all three time points in the antibiotics group, compared to 12 infants in the control group (p = 0.06, chi square test). This was similarly demonstrated in a previous strain-level analysis of infants’ gut microbiota that showed how colonization was interfered by antibiotics; species that are usually represented by a single strain, which indicates a single colonization event, were represented by several different strains, indicating multiple colonization events as a result of antibiotic treatment. [28]

Microbial recovery after antibiotic treatment in adults depends on the individual microbial resilience and is often incomplete.[22, 43] Since the microbiota of infants is not yet established,[44, 45] these effects cannot be expressed as a measure of return to initial state. Such perturbations may cause a permanent shift to an alternative state of the microbiota, as was described in adults,[43] or lead towards a different developmental trajectory.[13] Being a critical developmental phase, early microbiota perturbations may have dramatic long-term health effects, especially as it was recently shown that the timing and order of bacterial arrival is important to shape the gut microbiome.[46]

The fairly high number of healthy, term infants in this study, allowed thorough analyses of the progression of bacterial colonization in the gastrointestinal tract during the first three months of life. Additionally, our control group was homogenous by means of feeding type and delivery mode. However, this study also has several limitations. First, samples were obtained by the parents and stored in home freezers, so potential differences in storage may apply. Parents, however, were clearly instructed regarding collection and storage. Second, the IS-pro technique comprises two separate phylum-specific PCR reactions, which currently precludes direct quantification of Proteobacteria relative abundance against the other phyla. Also, the combination of multiple phyla into one group using common primers (FAFV), limits the interpretation of the results, especially concerning the low-Bacteroidetes settler type. Furthermore, incomplete information regarding antibiotics intake of the mothers during labor precludes the analysis of this potential confounder. Finally, a relative paucity of bifidobacterial species was found, which may be due to the techniques employed, such as DNA isolation protocols and PCR primers.[47, 48] While a more accurate representation of bifidobacterial species could contribute to establish further associations of settler types, or even enhance the observed antibiotics effect, it would not have an impact on the measurements of Bacteroidetes, based on which settler types were classified. However, it may still affect diversity analyses, and therefore also the dynamics of the settler types or treatment groups over time.

The pronounced effects of only one course of antibiotics, given as early as the infant is born, on the Bacteroidetes composition, and the confirmation of two distinct settler types that vary in Bacteroidetes abundance, suggest that certain infants may be more susceptible to the effects of antibiotics than others. This is also an indication that future studies analyzing similar perturbations of the microbiota should stratify infants based on their settler type. Classifying children into settler types, instead of delivery mode, may reveal group effects that might otherwise be masked and associations with disease later in life might prove easier to establish. Future studies should focus on establishing the presence and characteristics of these settler types in a larger infant population and assess factors associated with their development.

## Supporting information

S1 Fig Comparison of Bacteroidetes profiles of mother-child pairs from the Bacteroidetes-dominant settler type at 1 week of age (T = 1). Increasing intensity of the pink bars represent higher abundance of the species indicated.
(PDF)
