# Supplementary figures and images for: Neonatal microbiota development and the effect of early life antibiotics are determined by two distinct settler types
Source: PLoS One. 2020 Feb 5;15(2):e0228133. doi: 10.1371/journal.pone.0228133 (PMC7001974; doi:10.1371/journal.pone.0228133)

Bacteroidetes profiles of Child (C) Mother (M) pairs

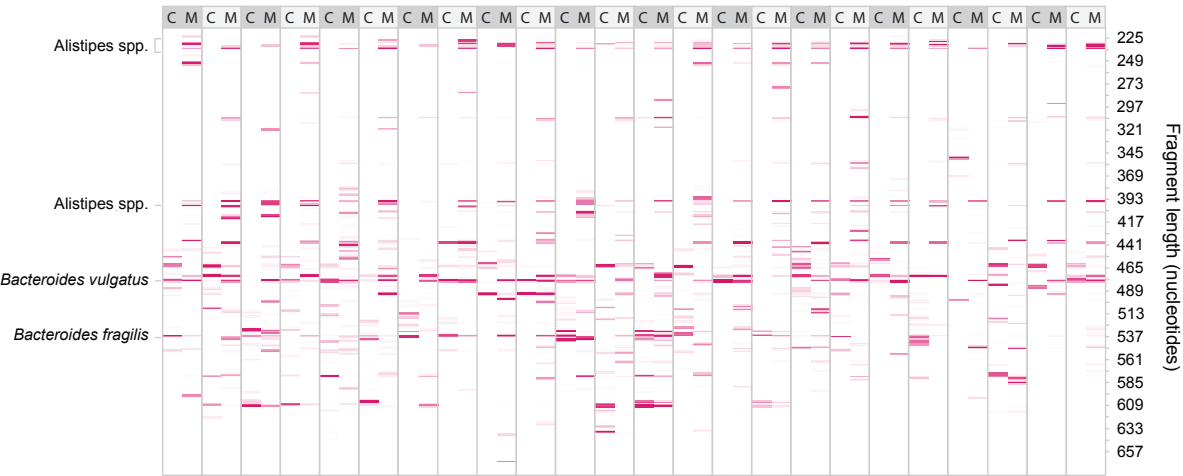

Supplement: S1 Fig — Increasing intensity of the pink bars represent higher abundance of the species indicated. (PDF) [file pone.0228133.s001.pdf]
